# Long intervals of stasis punctuated by bursts of positive selection in the seasonal evolution of influenza A virus

**DOI:** 10.1186/1745-6150-1-34

**Published:** 2006-10-26

**Authors:** Yuri I Wolf, Cecile Viboud, Edward C Holmes, Eugene V Koonin, David J Lipman

**Affiliations:** 1 National Center for Biotechnology Information, National Library of Medicine, National Institutes of Health, Bethesda, MD, USA; 2 Fogarty International Center, National Institutes of Health, Bethesda, MD, USA; 3 Center for Infectious Disease Dynamics, Department of Biology, The Pennsylvania State University, University Park, PA, USA

## Abstract

**Background:**

The interpandemic evolution of the influenza A virus hemagglutinin (HA) protein is commonly considered a paragon of rapid evolutionary change under positive selection in which amino acid replacements are fixed by virtue of their effect on antigenicity, enabling the virus to evade immune surveillance.

**Results:**

We performed phylogenetic analyses of the recently obtained large and relatively unbiased samples of the HA sequences from 1995–2005 isolates of the H3N2 and H1N1 subtypes of influenza A virus. Unexpectedly, it was found that the evolution of H3N2 HA includes long intervals of generally neutral sequence evolution without apparent substantial antigenic change ("stasis" periods) that are characterized by an excess of synonymous over nonsynonymous substitutions per site, lack of association of amino acid replacements with epitope regions, and slow extinction of coexisting virus lineages. These long periods of stasis are punctuated by shorter intervals of rapid evolution under positive selection during which new dominant lineages quickly displace previously coexisting ones. The preponderance of positive selection during intervals of rapid evolution is supported by the dramatic excess of amino acid replacements in the epitope regions of HA compared to replacements in the rest of the HA molecule. In contrast, the stasis intervals showed a much more uniform distribution of replacements over the HA molecule, with a statistically significant difference in the rate of synonymous over nonsynonymous substitution in the epitope regions between the two modes of evolution. A number of parallel amino acid replacements – the same amino acid substitution occurring independently in different lineages – were also detected in H3N2 HA. These parallel mutations were, largely, associated with periods of rapid fitness change, indicating that there are major limitations on evolutionary pathways during antigenic change. The finding that stasis is the prevailing modality of H3N2 evolution suggests that antigenic changes that lead to an increase in fitness typically result from epistatic interactions between several amino acid substitutions in the HA and, perhaps, other viral proteins. The strains that become dominant due to increased fitness emerge from low frequency strains thanks to the last amino acid replacement that completes the set of replacements required to produce a significant antigenic change; no subset of substitutions results in a biologically significant antigenic change and corresponding fitness increase. In contrast to H3N2, no clear intervals of evolution under positive selection were detected for the H1N1 HA during the same time span. Thus, the ascendancy of H1N1 in some seasons is, most likely, caused by the drop in the relative fitness of the previously prevailing H3N2 lineages as the fraction of susceptible hosts decreases during the stasis intervals.

**Conclusion:**

We show that the common view of the evolution of influenza virus as a rapid, positive selection-driven process is, at best, incomplete. Rather, the interpandemic evolution of influenza appears to consist of extended intervals of stasis, which are characterized by neutral sequence evolution, punctuated by shorter intervals of rapid fitness increase when evolutionary change is driven by positive selection. These observations have implications for influenza surveillance and vaccine formulation; in particular, the possibility exists that parallel amino acid replacements could serve as a predictor of new dominant strains.

**Reviewers:**

Ron Fouchier (nominated by Andrey Rzhetsky), David Krakauer, Christopher Lee

**Table 1:** Numbers of synonymous and nonsynonymous substitution per site (d_N_/d_S_) in H3N2 HA

Protein sites	d_N_/d_S _ratio; tree partition		
	All branches	Trunk branches	Other branches
H3N2 HA	0.27 ± 0.02	0.35 ± 0.08	0.26 ± 0.02
H3N2 HA1	0.37 ± 0.04	0.57 ± 0.15	0.34 ± 0.04
H3N2 HA2	0.13 ± 0.02	0.10 ± 0.05	0.14 ± 0.03
H3N2 epitopes	0.63 ± 0.09	1.85 ± 0.82	0.53 ± 0.08
H3N2 non-epitopes	0.15 ± 0.02	0.09 ± 0.04	0.16 ± 0.02

## Open peer review

Reviewed by Ron Fouchier (nominated by Andrey Rzhetsky), David Krakauer, Christopher Lee.

For the full reviews, please go to the Reviewers' comments section.

## Background

The antigenic variability of Type A influenza virus is the basis for the recurring epidemics that claim hundreds of thousands of human lives globally each year [1]. Unlike most pathogens where exposure leads to lasting immunity in the host, influenza A virus presents a moving antigenic target, evading specific immunity triggered by previous infections. This process, called *antigenic drift*, is the result of the selective fixation of mutations in the gene encoding the hemagglutinin (HA) protein, the major target for the host immune response[2]. Hemagglutinin variants that best escape the host immune response are thought to have a significant reproductive advantage [3].

Although less common than antigenic drift, *antigenic shift *is considered another major force in the evolution of influenza viruses[2,3]. Antigenic shift occurs when the virus acquires an HA of a different influenza subtype via reassortment of one or more gene segments and is thought to be the basis for the more devastating influenza pandemics that occurred several times in the last century[4]. There have been three pandemics in the last hundred years: in 1918 (H1N1 subtype), 1957 (H2N2 subtype), and in 1968 (H3N2 subtype). During each of these pandemics, the new virus drove the previous pandemic subtype out of circulation. In 1977, the H1N1 subtype reappeared, albeit with a lower virulence than both the original H1N1 of the 1918–1956 period and the H3N2 subtype, and since then has been co-circulating with H3N2 [3,4].

In some seasons, the Type A influenza cases are primarily due to H3N2, the predominant subtype circulating in humans since 1969. In other seasons, however, the H1N1 subtype predominates (c.f. [5]). Reassortment of influenza gene segments also occurs *within *co-circulating lineages of the same subtype, providing an additional mechanism for generating diversity during the interpandemic evolution of the virus [6,7]. In particular, intra-subtype reassortment was responsible for generating the 2003 Fujian-type antigenic strain [8,9], demonstrating the epidemiological significance of this phenomenon.

The observations of extremely rapid evolution of influenza A, especially in the HA gene, has led to the suggestion that the evolution of the surface regions of the HA is driven by continual positive selection. In particular, Ratner et al., in a phylogenetic analysis of human H3 subtype HA genes, found a significantly higher rate of amino acid replacements than silent changes in antigenic positions of the HA1 domain (which consists of the N-terminal 329 residues of HA and includes the epitopes recognized by the immune system) as compared to the same rates (or rate ratios) for the C-terminal HA2 domain [10]. These findings of apparent positive selection in the HA gene were further supported and extended in similar studies by Fitch et al. [11] and Ina and Gojobori [12]. It has been emphasized that the phylogenetic tree for the HA1 of H3N2 isolates has a distinct, "ladder-like" shape, with a prominent trunk (the path from the root to the base of the latest included cluster of isolates) and, typically, short side branches [11].

More recently, Bush et al. performed an in-depth study of 357 nucleotide sequences of the HA1 domain of the HA gene from human H3N2 subtype influenza virus isolated between 1983 and 1997 [13]. This study supported earlier findings of positive selection for those amino acid positions involved in receptor and antibody binding and, more specifically, for a subset of 18 amino acid sites in HA1. In addition, Bush et al. found that 9 of these 18 positions showed evidence of positive selection only in the internal branches and not the terminal branches (i.e. the tips) of a phylogenetic tree generated from the entire set of HA1 sequences. Given this bias, it has been proposed that these positions would be useful in predicting future dominant epidemic variants [13]. This, and other efforts to improve prediction of epidemic strains [14] are deemed important for improving influenza vaccine formulation because the closer the vaccine strain is to the dominant variant, the more effective the vaccine [3].

Most influenza gene sequencing is done as part of international surveillance programs whose focus on identifying serologically novel strains results in biased samples of the viral population [14,15]. The Influenza Genome Sequencing Project, funded and managed by the US National Institute of Allergy and Infectious Diseases, has recently generated over 1000 fully sequenced influenza genomes from clinical isolates obtained between 1995 and 2005 and publicly available in Genbank [16]. One goal of this project was to provide researchers with a large set of sequenced isolates that represent a relatively unbiased, i.e., not enriched in antigenically novel isolates, view of influenza strains in the population [17]. We analyzed this data set along with other currently available H3N2 and H1N1 sequences to further refine our understanding of the interpandemic evolution of influenza A virus, the relative role of positive selection versus random genetic drift in HA, and the implications for epidemic surveillance.

## Results

**Figure 1:**
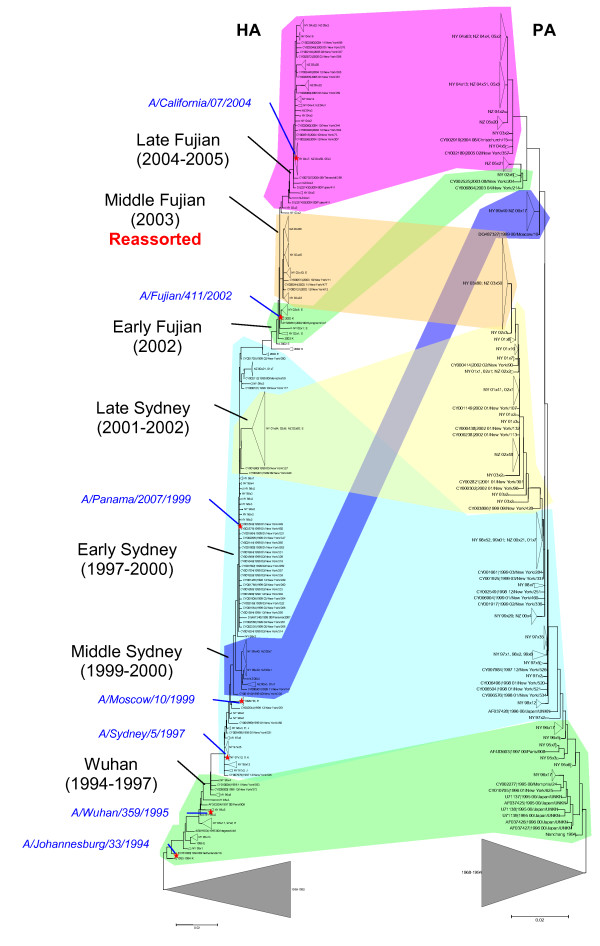
A comparison of the phylogenetic trees for the HA and PA genes of the H3N2 subtype of influenza A virus from 1994–2005. The colored connectors indicate major discrepancies between the gene trees that result from reassortment. The subtrees corresponding to sequences from isolates prior to 1994 are collapsed to focus on isolates from 1994 through 2005 and portions of the trees are labeled ("Wuhan", "Middle Sydney", etc.) with names derived from antigenically distinctive isolates that dominated during particular time intervals. Approximate positions of the vaccine isolates are indicated by red asterisks and blue, italicized labels.

Figure 1 shows juxtaposed evolutionary trees of human H3N2 HA and PA (polymerase) genes, with colored shading connecting HA and PA tree partitions comprised of gene segments from the same clinical isolate. The PA gene was chosen as a control to the HA because it encodes an internal viral protein and is among the viral genes that are the least likely to be subject to substantial positive selection. As is generally seen in similar comparisons, gene segments roughly cluster by the sampling date. However, the temporal ordering of the Sydney isolates (1997–2002) is somewhat indistinct, with, for example, the dominant isolates of 2001 being derived from 1998 isolates rather than the dominant isolates of 2000 or 1999. Associated with this is a major branching of the PA tree with one subtree comprised of isolates primarily from 2001–2003 and another subtree with isolates from 1999 and 2002–2005.

While the HA and PA trees are generally consistent, the lack of clear temporal succession in the HA tree during the "Sydney" antigenic period, along with the bifurcation of the PA tree, results in a HA/PA inconsistency, or apparent "crossing" for isolates from 1999–2000 (Figure 1)). A more dramatic inconsistency, however, is seen in the Fujian antigenic interval associated with the reassortment noted by Barr et al. and Holmes et al. [8,9]. As seen in Figure 1, the Fujian strain was first observed in Europe and Southeast Asia in 2002 and in New York state in April, 2003, which was an H1-dominant influenza season in the US, and appears more closely related to dominant strains from 1998 (denoted here as "Early Sydney") than, for example, to strains from 2001–2002. The reassortment event generated a strain with the Early Fujian HA gene segment, but all other gene segments derived from a Late Sydney strain and this reassortant becomes the dominant strain in 2003–2004 season. Notably, however, this reassortant strain is completely replaced by the non-reassorted Fujian strain ("late Fujian") from 2004 onwards. This observation has not been made by Barr et al or Holmes et al. [8,9] because the relevant data was not available at the time when these analyses were conducted.

To determine whether positive selection played a substantial role in the evolution of the HA gene during the period covered by the isolates analyzed, we computed the ratio of nonsynonymous (d_N_) to synonymous (d_S_) nucleotide substitutions per site (ratio d_N_/d_S_) for (i) all branches of the H3N2 HA tree, (ii) for the branches comprising the trunk lineage only, and (iii) for all non-trunk branches (Table 1). The results were further subdivided to consider mutations anywhere in the HA protein, or restricted to the HA1 domain, the HA2 domain, the epitope regions of the HA1, and the non-epitope regions of the HA1 (see Methods). In agreement with previous work [10-13,18], there was a consistent pattern such that the d_N_/d_S _ratio was considerably higher in the regions of the protein recognized by the host immune system (i.e., epitopes > HA1 > HA2) and in the trunk lineage (i.e., the mutations transmitted to the subsequent generations). These differences were not statistically significant but this is, most likely, due to the small number of substitutions in the currently available data. Further, only the d_N_/d_S _value for the trunk mutations in the epitope regions was >1, which is considered to be indicative of positive selection [19]. Although this result was not statistically significant, most likely, again because of the small number of mutations in the trunk, it strongly suggests that positive selection only affected the H3N2 trunk lineages and only the epitope regions of HA.

**Figure 2:**
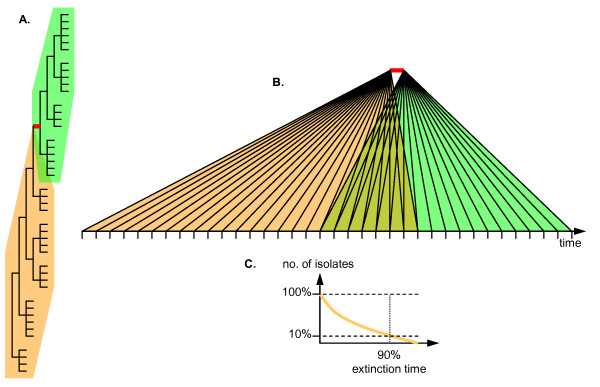
Calculation of extinction times. A. An arbitrary trunk branch (highlighted in red) divides a tree into ancestral (orange shading) and descendant (green shading) parts. B. Terminal nodes (isolates) are arranged along the time axis; the overlap area denotes the period of time during which the descendant lineages drive the ancestral lineages to extinction. C. Extinction curve of the isolates from the ancestral part of the tree, for the overlap interval shown in (B); the 90% extinction point is indicated.

**Figure 3:**
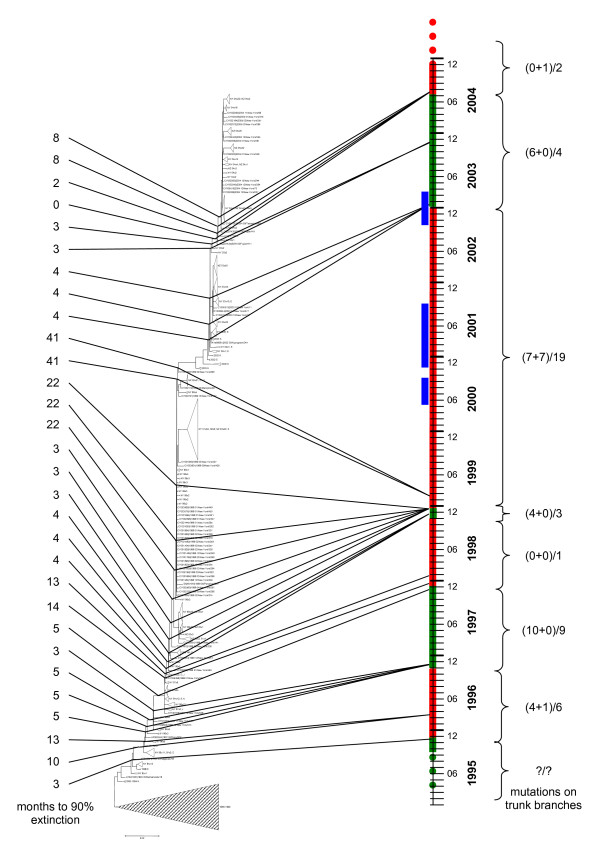
Periods of stasis and rapid fitness change in the evolution of H3N2 HA. Red intervals indicate stasis and green intervals indicate rapid change. Blue bars show the intervals of H1N1 dominance. The numbers to the left of the tree indicate the extinction time of the co-existing descendants of the given node. The ratios to the right are: (nonsynonymous mutations in epitopes + nonsynonymous mutations outside epitopes)/synonymous mutations in the trunk branches.

Because the sample of clinical isolates in the NIAID Influenza Genome Sequencing Project is approximately unbiased – antigenic variants are not preferentially selected – the relative frequencies of H3N2 lineages over time are expected to directly reflect fitness differences. Using the HA tree from Figure 1 as a guide, we divided the H3N2 isolates into distinct lineages based on the trunk branch from which they derive (Figure 2). We can then assign an origination date to each lineage based on the earliest date of any isolate within the group; although this is a conservative estimate, it is consistent across all lineages. With these assignments of lineages and their origination dates, we can compare the extinction times of successive lineages as shown in Figure 3 (see Methods for details). This analysis reveals a sharp distinction between lineages with short (<6 months) extinction times (green intervals in Figure 3) and those with long (>6 months) extinction times (red intervals in Figure 3). The most likely explanation of this pattern is that new lineages with greater advantages in fitness more rapidly and fully drive older co-circulating lineages to extinction than new lineages with minimal or no fitness advantages. The latter lineages tend not to exterminate previously circulating isolates such that these can reappear in later seasons. Compatible with this explanation, the intervals of rapid extinction (fitness change) are associated with an excess of nonsynonymous over synonymous substitutions, with the former occurring almost exclusively in the epitope regions (Figure 3). Specifically, the ratio of the number of amino acid replacements in epitopes to the number of replacements in non-epitope regions was 23:1 for the sum of the rapid extinction intervals (green) and 9:8 for the sum of the slow extinction intervals (red). The difference between these ratios was statistically significant, with P = 0.0017 by Fisher's exact test. Similarly, the ratio of nucleotide substitutions leading to amino acid replacements in the epitopes to all other substitutions (both synonymous and nonsynonymous) was 23:24 for the "green" intervals and 9:30 for the red intervals (P = 0.012 by Fisher's exact test). These findings strongly suggest that the rapid extinction of virus lineages is driven by positive selection in the epitope regions of each new dominant lineage, whereas the intervals of slow extinction (defined here as "stasis" periods when no major antigenic changes occur) largely involve neutral or near-neutral evolution.

**Figure 4:**
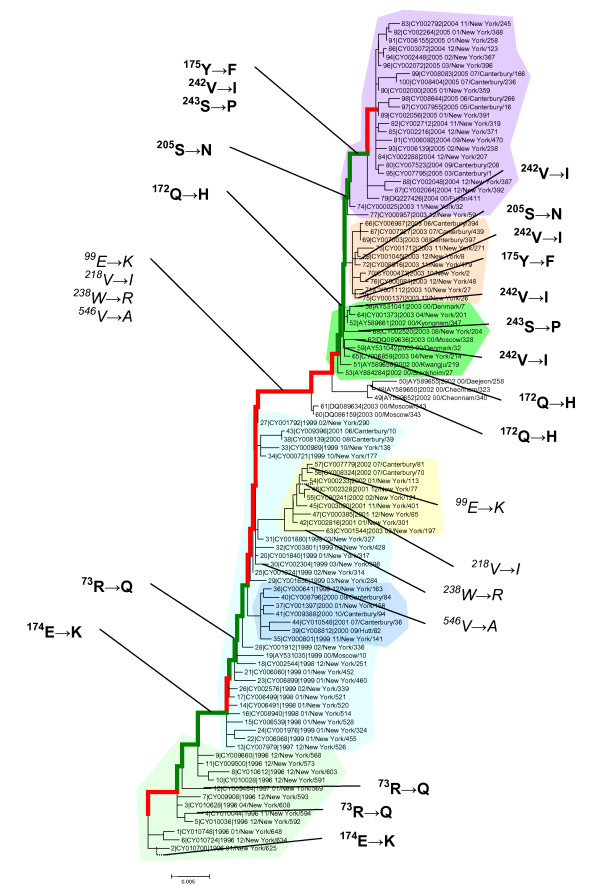
Parallel amino acid replacements in H3N2 HA. The replacements in epitope regions are shown in boldface. Color-code for shading of lineages is as in Figure 1. Stasis intervals are indicated in red in the main trunk of the tree, while intervals associated with rapid fitness change appear in green, as in Figure 2.

An additional distinction between the stasis intervals and intervals of rapid fitness change became apparent through the analysis of parallel mutations in HA, i.e., the mutations that occur on the side tree branches in parallel to those occurring on the main trunk of the tree in approximately the same time period. Altogether, for the 1995–2005 time interval, parallel replacements were detected in 11 positions of the HA protein (Figure 4). Notably, 7 of these positions were located in the epitope regions and nearly all the mutations in these positions mapped to the putative positive selection intervals discussed above (the green intervals in Figures 3 and 4). Two of these positions harbor three parallel mutations each (HA positions 73 and 172) and one position – 242 – has five parallel mutations. Our observation of the same mutations recurring in multiple lineages is compatible with the notion that each confers a substantial selective advantage onto the respective lineage, and there are limited fitness trajectories available to viral evolution. This conclusion reverberates with the results of recent studies on experimental evolution of enzyme fitness which demonstrated that, among the multitude of possible mutational trajectories, only a small fraction is accessible to selection [20]. In contrast, the only parallel mutations that exclusively occurred in periods of stasis (red intervals) were also the only ones to occur in non-epitope regions of the HA protein (Figure 4).

In an attempt to obtain independent evidence of periods of "stasis" in influenza evolution, we examined long-term epidemiological records, namely, the weekly time series of Pneumonia and Influenza (P&I) deaths from 1972 to 2002 in US states ([21]). We computed the influenza-related mortality impact for each season as the number of P&I deaths above a seasonal baseline. We also calculated a measure of regional speed of spread for each epidemic, based on the timing of P&I peaks in each state. The predominant virus subtype was defined for each season by CDC laboratory surveillance as the subtype(s) representing >75% of influenza specimens collected in the US. A pattern compatible with stasis was observed in the H3N2 seasons preceding an H1N1 season: these seasons were associated with significantly slower spread than other H3N2 seasons (spread index = 5.69 weeks versus 3.60, P = 0.02 by the Wilcoxon test), and a somewhat lower mortality impact (3.32 P&I excess deaths per 100,000 population versus 4.55, Wilcoxon P = 0.08). Overall, H1N1 seasons were associated with lower mortality impact and slower spread than H3N2 seasons (mortality index: 1.8 P&I excess deaths per 100,000 in H1N1 seasons versus 4.1 in H3N2 seasons, Wilcoxon P = 0.005; spread index: 7.1 weeks versus 4.4 weeks, P = 0.008). This suggests that H1N1 is able to out-compete H3N2 only when the epidemiologic fitness of H3N2 lineages declines below a certain level.

**Figure 5:**
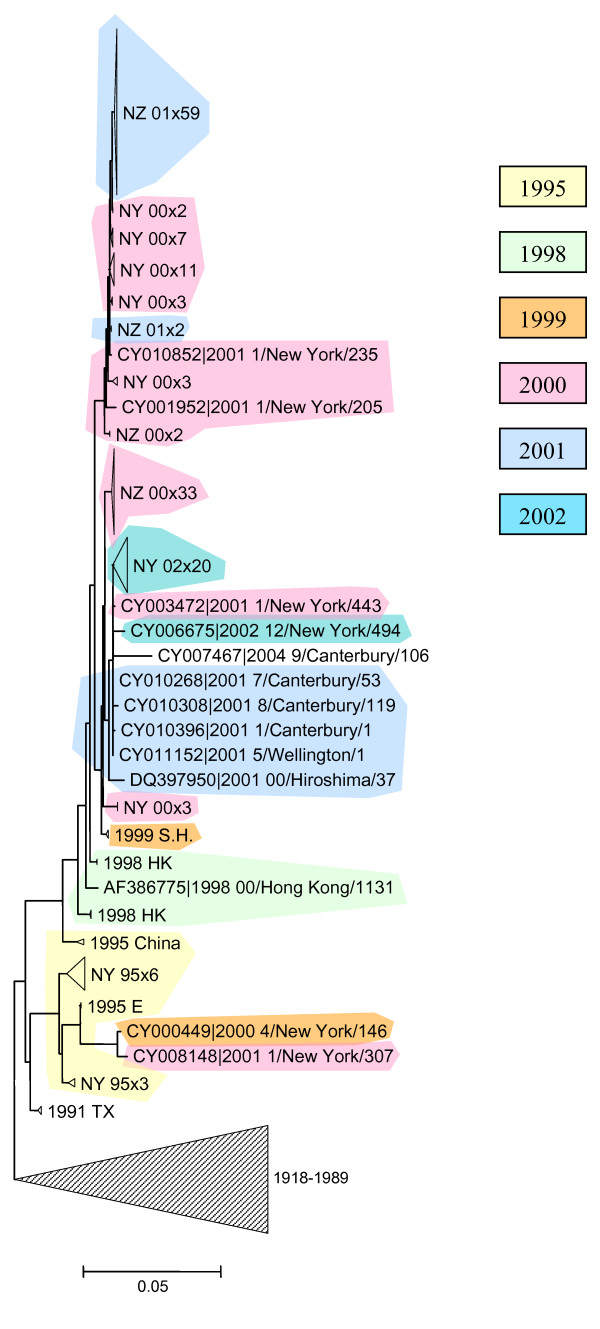
Prolonged stasis in the evolution of H1N1 HA. Isolates from the same influenza season are shaded in the same color.

As noted above, most influenza seasons are dominated by the H3N2 subtype whereas, since 1977, some other seasons were dominated by the milder H1N1 subtype. Immune cross-protection between different subtypes of influenza A has been observed [22,23] such that there is competition between H3N2 and H1N1. During the long stasis interval spanning 1999–2002, there were two H1N1-dominant seasons in the US, 2000–2001 and 2002–2003 [5]. Consistent with the surveillance results, we observed a preponderance of H1N1 isolates in both the New York and the New Zealand data sets during several intervals between 2000 and 2003 (Figure 3). The transition to H1N1 could be due to either positive selection leading to increased fitness of H1N1 itself, or to a relative decrease in H3N2 fitness as a greater proportion of the host population had been exposed to a relatively static set of H3N2 strains. The d_N_/d_S _analysis of the H1N1 tree, including the portion corresponding to this interval, showed no evidence of positive selection (Table 2), and this was also the case when d_N_/d_S _ratios were computed for each codon independently (data not shown). In accord with these observations, there was no pattern of lineage displacement in the H1N1 tree (Figure 5). These results are consistent with a picture of the general neutral evolution of the H1N1 strains and suggest that H1N1 took over only due to the drop in the relative fitness of H3N2.

**Table 2:** Numbers of synonymous and nonsynonymous substitution per site (d_N_/d_S_) in H1N1 HA

Protein sites	d_N_/d_S _ratio; tree partition		
	
	All branches	Inter-clade branches*	Other branches
H1N1 HA	0.20 ± 0.04	0.15 ± 0.08	0.21 ± 0.04
H1N1 HA1	0.22 ± 0.05	0.19 ± 0.12	0.22 ± 0.05
H1N1 HA2	0.15 ± 0.05	0.08 ± 0.09	0.17 ± 0.06

*refers to several branches of the H1N1 HA tree that connect the 2000–2001 clade and the 2000–2004 clade; unlike H3N2, the trunk is not identifiable in the H1N1 tree within the analyzed time interval.

## Discussion

Most studies on the interpandemic evolution of influenza virus have focused on antigenic drift, in which mutations in the epitope regions of the HA protein are thought to be highly favored if they allow the virus to escape the host immune system [3,11-13]. Under this view, evolution of the influenza virus HA is, largely, driven by positive selection.

The results described here suggest a very different picture of HA evolution. Indeed, the most salient feature of H3N2 evolution during the 1994–2005 time interval appears to be the predominance of neutral sequence evolution manifest in extended periods of antigenic stasis that are not associated with major fitness change. During these stasis periods, there is a preponderance of silent nucleotide changes, and those amino acid replacements that do occur are not preferentially located in the antigenic regions of the hemagglutinin, suggesting that they are neutral. Thus, these amino acid replacements do not seem to affect the antigenic properties of HA to the extent that would allow the respective viruses to evade the immune system and, consequently, are not favored by natural selection. Consistent with the excess of neutral mutations during the intervals of stasis, displacement of pre-existing lineages by new strains is slow, again suggesting that they only differ minimally in fitness.

We therefore propose that, during periods of stasis, any antigenic novelty is insufficient to yield substantial fitness advantages for competing H3N2 strains. Consequently, the absolute fitness of the H3N2 variants drops as the density of susceptible hosts gradually decreases. Hence, although the H1N1 subtype also seems to be evolving neutrally, its relative fitness increases as an increasing number of individuals who have not been exposed to H1N1 enter the population. Ultimately, the fitness trajectories of the H3N2 and H1N1 subtypes cross, such that H1N1 was able to out-compete H3N2 in 2000 and 2002. Although we do not have sufficient sequence data to extend this analysis prior to 1995, the epidemiologic observations of decreased speed of spread and, possibly, decreased excess mortality for H3N2-years immediately prior to H1N1 years suggest that periods of stasis have always been a feature of the interpandemic evolution of the H3N2 subtype.

The intervals of stasis in influenza A virus evolution are punctuated by periods of apparent rapid change in fitness (owing primarily to antigenic innovation), which are associated with an excess of nonsynonymous mutations in epitope regions of HA and the rapid displacement of old lineages by new dominant ones. The high frequency of parallel mutations during intervals of rapid fitness change indicates both that the virus is able to rapidly explore the adaptive landscape, fixing antigenically favorable mutations, and that there are a limited number of pathways across the adaptive landscape. Thus, the observation that the H3N2 virus was in stasis for most of the 1995–2005 time interval suggests that for much of this time the HA gene was more than a single mutational step away from a significant increase in fitness. In other words, from a given HA sequence, several mutations seem to be required to yield an antigenically distinct HA, and little or no fitness advantage is conferred by any subset of these mutations – a form of epistasis. Consequently, new dominant strains are likely to often emerge as a result of a single mutation of pre-existing low frequency (and low fitness) strains, a pattern that is clear from examination of Figure 1.

Compelling evidence for this type of epistatic interaction between amino acids in the influenza hemagglutinin has been obtained in a study on the effect of HA mutagenesis on its hemabsorption properties [24]. Consistent with this, Smith et al. noted that some mutations caused much greater antigenic change than others, i.e., that the long-term rate of antigenic change was less uniform than the rate of genetic evolution [18]. A major role of synergistic epistasis in virus evolution of has been convincingly demonstrated also for other RNA viruses including HIV [25,26] and vesicular stomatitis virus [27]. From a more general evolutionary standpoint, these results are consistent with the notion that neutral mutations provide essential material for subsequent evolutionary innovation [28-31].

The great majority of influenza isolates analyzed here come either from New York State or from New Zealand, and in particular, all the results on extinction times were obtained with isolates from these locations. Thus, the sample is geographically limited, although both the Northern and the Southern hemispheres are represented. However, the observation on extended periods of stasis described here is highly unlikely to be an artifact of incompleteness in the data, especially as the genetic diversity of influenza virus in New York State is representative of that on the global scale [9] and that viruses sampled from New York State or New Zealand are intermingled on our phylogenetic trees. Moreover, recently, it has been shown that epidemics in New York State are seeded by the seasonal importation of multiple lineages of influenza viruses, rather than local strains persisting during the summer, suggesting that this dataset provides a good sample of the global diversity of influenza viruses [32]. Additionally, such a hypothetical, "missing", positively selected branch would have had to go extinct despite its high fitness, otherwise it would appear in our analysis of the trunk isolates.

The observations described here, along with several previous ones, suggest that interpandemic evolution of influenza virus is a highly complex, multifaceted process in which reassortment, as observed in the emergence of Fujian-like viruses [8,9], played an important role. Critically, however, our results indicate that viral fitness does not solely depend on antigenic novelty. A shown in Figure 1, the Fujian strain first appeared in 2002 from a low frequency predecessor that first diverged from the Sydney strain, probably, in 1998. The experiments of Jin et al [33] indicate that the amino acid replacement in position 171 resulted in a major change in antigenicity from the then prevailing strains, and this change was further enhanced by the replacement in position 172. The three parallel mutations observed in position 172 (Figure 4) corroborate the selective value of this replacement and are consistent with the experimental results. However, the antigenic novelty of the 2002 early Fujian strain notwithstanding, it was only a minor H3N2 variant during an H1N1 dominant season. That the HA of this virus was able to confer a significant fitness advantage onto the reassortant virus suggests that the early Fujian strain carried deleterious mutations elsewhere in its genome that counteracted the fitness advantages from the antigenically novel HA. By 2004, however, the "pure" Fujian had out-competed the reassortant virus, most likely because of subsequent compensatory mutations. It is also conceivable that the reassortant, although benefiting from the antigenic novelty of the Fujian HA gene, also suffered some disadvantages or incompatibilities. For example, it has been suggested that the HA and NA of the reassortant had mismatched hemagglutinin and neuraminidase specificities [34].

These results have implications for influenza surveillance and vaccine formulation. Accurate prediction of the dominant strain during intervals of rapid fitness change is expected to be extremely challenging. Since fitness differences among strains are small during stasis, there could be many genetically distinct clades at low frequency in the population that are initially indistinguishable by serological surveillance. While we do not currently have methods to predict which of these variants are one mutational step away from an antigenically distinct descendant, there is ample evidence that these new variants can rapidly become dominant (e.g., Figures 1 and 3). Presumably, this was the cause of the vaccine mismatch for the 1997 influenza season which saw the emergence of the Sydney strain whereas the vaccine recommendation was for a Wuhan H3N2 strain [35]. The Fujian strain also emerged from a low frequency clade as discussed above but, perhaps, because of deleterious mutations elsewhere in the genome, it was not initially dominant. Although the antigenic distinctiveness of Fujian had been noticed [36], this strain grew poorly in eggs, thus presenting a challenge to incorporate in the vaccine. Since a significant number of the H3N2 isolates at that time were still of the Late Sydney variant, the Sydney strain was retained for vaccine use [36], leading to a mismatch with the Fujian reassortant that dominated that year.

Matching the vaccine strain to the dominant strain is particularly challenging during periods of rapid antigenic change. Furthermore, some of these intervals can be extremely short, with only a small number of amino acid replacements (e.g., the short green interval in the 1998–99 season, with only 4 replacements, shown in Figure 1) although they are nevertheless associated with significant changes in fitness. As a case in point, Hardy et al. [37] and Schweiger et al. ([38] noted new variants becoming dominant during the 1998–1999 season that were distinguishable genetically but not serologically. Because the hemagglutination inhibition (HI) assay was unable to detect serological novelty with this new variant, the vaccine strain choice for the 1999–2000 season was again an early Sydney strain [39]. Thus, although, on average, the HI test is a reasonable surrogate for the human immune response, there might be cases when this test misses epidemiologically relevant serological differences between influenza isolates.

Consequently, strains that have accumulated amino acid replacements in epitopic regions and have been shown to quickly displace co-circulating strains seem to merit attention as potentially epidemiologically significant even in the absence of indications from the HI test. This study suggests particular retrospective cases in this category that might be worth additional, detailed serological examination.

Correct prediction of the dominant strain is difficult even for periods of stasis because of the temporal indistinctness of the isolate succession (see above). However, the consequences of a mismatch during these intervals are likely to be less dramatic given the absence of major antigenic changes.

Considering that the stasis intervals allow the proliferation of low frequency clades, any of which might become the next dominant strain, sequencing much larger numbers of representative isolates should be helpful in augmenting current surveillance methods. A clade that persisted for several years at low frequency might warrant further characterization, especially, considering the history of the Fujian strain. Furthermore, more intense sampling would allow the detection of additional cases of parallel amino acid replacements, which might be the earliest sign that certain mutations are being fixed by selection and could soon provide significant fitness advantages. To evaluate potential benefits of deeper sampling of influenza isolates by genomic sequencing, it will be important to sequence a large number of geographically dispersed isolates from 1996 to determine whether an increase in parallel mutations presaged the 1997 Sydney epidemic.

Taken together, the findings described here indicate that interpandemic evolution of influenza A virus involves a complex interplay between neutral evolution during periods of antigenic stasis, positive selection during relatively short intervals of rapid change in fitness, and multiple effects of reassortment. It is notable that analysis of a relatively large and unbiased sample of viral sequences from multiple seasons with straightforward molecular evolutionary methods yields this unexpectedly complex picture of virus evolution. To further elaborate and complete this picture, additional, large-scale sequencing of diverse influenza virus isolates is critical.

## Materials and methods

### Sequence data

Our analysis utilized human Influenza A virus data retrieved from the NCBI Influenza Virus Resource [16]. All (nearly) full-length protein and coding region (CDS) sequences (550+ amino acids for HA and 714+ amino acids for PA) were downloaded and aligned using the MUSCLE multiple alignment program [40]. CDS sequences were aligned to match the protein alignments codon-by-codon. The resulting alignments included 227 H1N1 HA sequences, 994 H3N2 HA sequences and 894 H3N2 PA sequences. The Genbank accession numbers for all analyzed sequences are available as Supplementary Material (see Additional File 1)

### Phylogenetic analysis

Maximum parsimony trees were reconstructed from the CDS alignments using the PAUP* program [41] utilizing subtree pruning-regrafting (SPR) branch-swapping. 50% majority rule consensus trees (with zero-length branches collapsed) were used to represent the evolutionary history of each segment. Each tree was rooted at the cluster of sequences from the oldest available isolates (1918 for H1N1 and 1968 for H3N2). The "trunk" of the tree was defined as the path from the root to the base of the cluster including the latest (2005) sequences. Mutations were mapped to individual tree branches using the DNAPARS program of the PHYLIP package (with consensus maximum parsimony trees supplied to DNAPARS) [42,43].

### Analysis of selection pressures

To examine the selection pressures acting on HA we used a subset of recent (1995–2005) sequences representing all distinct side branches that were selected using the complete maximum parsimony tree as a guide to the phylogenetic relationships between the isolates. The selected sets included 100 H3N2 sequences and 20 H1N1 sequences. A subtree joining the selected sequences, was extracted from the full-size tree and used as input for the CODEML program of the PAML package [44,45]. CODEML was then used to obtain maximum likelihood estimates of the d_N_/d_S _ratio per site for the whole tree (basic model [46]) or independently for subsets of branches (e.g. trunk vs. non-trunk branches, branch model [47].

In addition to the full-length HA sequences, this analysis was performed with the alignment partitioned into the HA1 and HA2 segments and into antigenic epitope and non-epitope sites [48,49]. The sequence coordinates of the epitope positions were from [48,49] which were themselves derived from the crystal structure of the HA of a 1968 Hong Kong isolate of H3N2 (A/Aichi/2/1968) reported by Wiley et al. [48,49]. These coordinates were then projected onto the multiple alignment of the HA protein sequences (see Additional File 1).

Maximum likelihood reconstructions of ancestral sequences and individual mutation events, provided by PAML, were used to cross-validate the maximum parsimony reconstructions.

To estimate the selection pressures acting on the HA gene from H1N1 in more detail, we inferred codon-specific d_N_/d_S _values using the Single Likelihood Ancestor Counting (SLAC), Fixed Effects Likelihood (FEL) and Random Effects Likelihood (REL) methods, all incorporating the HKY85 substitution models with phylogenetic trees inferred using the Neighbor-Joining method available at the Datamonkey facility [50]. No evidence for positive selection in H1N1 was found under any method.

### Extinction of lineages and the tempo and mode of influenza evolution

Each trunk branch divides the tree (and the set of the terminal nodes) into two parts: one "below" this branch (i.e. towards the root) and the other one "above" (Figure 2). Terminal nodes (tips) associated with the proximal partition of the tree represent isolates whose lineages are bound for extinction; the tips in the distal partition represent isolates descending from the breakpoint branch. The oldest isolate in the descendant partition of the tree provides the upper bound time estimate for the breakpoint branch (obviously, the breakpoint branch must predate the oldest descendant node). The youngest isolate in the extinct partition of the tree can survive well past the breakpoint. Proximal partition isolates that are younger than the breakpoint represent the lineages co-existing with the descendant lineages for some time. The process of extinction of these lineages can be described in terms of the fraction of the lineages that survive past the given time interval from the breakpoint. Here, we associate each of the trunk branches with the amount of time that is required for 90% extinction (10% survival) of the co-existing lineages (Figure 2); similar results were obtained with 50% or 75% extinction thresholds. Only isolates with precisely known isolation dates, mostly, those from the NIAID-funded projects in New York State, USA, and New Zealand, were used in this analysis. It is reasonable to assume that rapid displacement of the existing lineages by the descendants of a particular trunk branch indicates a highly competitive evolutionary landscape with the isolate replacement driven by positive selection. By contrast, slow extinction indicates little (if any) difference in fitness between the co-existing lineages from the proximal and distal partitions of the tree, i.e., a (nearly) neutral mode of evolution.

## Reviewers' comments

### Reviewer's report 1

**Ron Fouchier**, National Influenza Center and Department of Virology, Erasmus Medical Centre, Rotterdam, The Netherlands (nominated by Andrey Rzhetsky)

In the manuscript, in particular in the abstract, the authors refer to phenotype changes while the phenotype is only INFERRED from genetic data. For instance, antigenic changes are inferred from amino acid replacements in proposed epitopes, and changes in virus fitness are inferred from rapid displacement of existing lineages. It would be better to stick to the facts; e.g. refer to amino acid substitutions in antigenic sites rather than antigenic change and to use the term fitness with more care.

**Author response: ***Antigenic changes are not, exactly, inferred from changes in the (proposed) epitopes but, of course, when we notice differential patterns for replacements in the epitope regions, we believe that it makes sense to interpret these differences as being relevant to the evolution of the virus antigenicity and fitness. We went through the corresponding language throughout the paper and made minor changes, in particular, in the Abstract, to emphasize that we are talking about "apparent" antigenic changes. As far as viral fitness is concerned, there is no inference involved inasmuch as the rate of displacement of pre-existing lineages is what, actually, defines fitness. Thus, apart from the aforementioned small modifications, we believe that we have been sticking to the facts to begin with*.

**Background**, 4^th ^paragraph, and **Results**, 3^nd ^paragraph, and **Methods **section Please specify the "antigenic positions of the HA1 domain". The paper by Ratner et al. is in Russian, which is not a language that everyone is familiar with. The method section further refers to a statistical analysis and a structural analysis, without providing us with a list of positions that are considered to represent epitopes. It is tricky to take either of these papers as the gold standard for where the epitopes are located. It would thus be good to list the "antigenic positions" in HA1 here.

**Author response: ***The problem here is that the positions of the epitopes in the particular isolate for which they have been reported were projected on the multiple alignment of the HA sequences. Thus, the specific coordinates in that particular isolate would hardly help much. In the revision, we expanded the explanation of the procedure under Methods (section on **Analysis of Selection Pressures**, 2^*nd *^paragraph). The relevance of Ratner's paper here is unclear as it is not about antigenic positions per se but about evidence of positive selection in HA. We felt that it was highly desirable to cite this paper as the first one (to our knowledge) that presented such evidence. It is available in English translation*.

**Background**, last paragraph, **Results**, 4^th ^paragraph, and **Discussion **section on "bias" While I fully agree that the influenza sequence databases are heavily biased towards "outlier" strains (with unusual antigenic properties as compared to other viruses isolated during the same epidemic), it is incorrect to assume that the sequence collection analyzed here is not biased. First of all, the sequences are primarily from short epidemics in New York State and New Zealand. With a virus that circulates (and evolves) globally, this bias has large implications for the type of work described here. In addition to geography, there may be several other biases (sampled patient populations, inclusion criteria for the sequencing project, etc.) that could affect the fitness discussions that follow. The authors have not addressed this issue at all, and simply assume that their dataset is good enough. I am not so sure.

**Author response: ***This is, indeed, an important issue, and we agree that we did not address it in sufficient detail in the original version. However, there are relatively straightforward arguments to the effect that geographical or other bias is extremely unlikely to account for the pattern of stasis-rapid change that we consistently observe in influenza evolution. These arguments are presented in the revision (Discussion, 6^*th *^paragraph)*.

**Results**, 2^nd ^paragraph:

It is not clear to me what the results described here add to the information provided in the papers by Barr et al., and Holmes et al. that described the reassortant viruses in detail (refs [7] and [8]). Could thus be shortened.

**Author response: ***Surely, these papers have described the reassortant viruses. However, a critical facet is missing there, namely, the demonstration that "...reassortant strain is completely replaced by the non-reassorted Fujian strain ("late Fujian") from 2004 onwards" (quote from our revised paper, Results, 2^*nd *^paragraph; so this clarification was added*.

**Results**, 2^nd ^paragraph from the end:

Only data from the US are included, when the authors state that 2000–2001 and 2002–2003 were H1N1 seasons. While this may be true in the US, it certainly was not true for many other parts of the world. Should the competition between fit and less fit variants of H3N2 viruses and those of the H1N1 subtype not be considered at the global scale? Surely, with influenza viruses moving from Northern hemisphere to Southern hemisphere and back with the seasons, the viruses evolve and compete on a global scale, not just in the US.

**Author response: ***The pattern was the same for New York State and New Zealand which is shown in the revised Figure *3. *We do not have comparable depth of data for other parts of the world, unfortunately*.

### On the analyses of H1N1 strains (results and discussion)

The authors indicate that there was no positive selection in H1N1. Despite the apparent lack of positive selection, there have been vaccine updates in H1N1 strains because of antigenic drift between 1995 and 2005 (e.g. A/New Caledonia/2000). The question than arises; Do we need positive selection for antigenic drift to occur? If not, we cannot rely on sequences to identify when vaccine updates are necessary?

**Author response: ***We never claimed that antigenic drift strictly requires positive selection. What we do claim is that there is no evidence of substantial positive selection in H1N1 during the analyzed time interval, and accordingly, the dominance of H1N1 in some seasons was caused by the lack of highly fit H3N2 isolates rather than by emergence of novel and highly fit H1N1 viruses. In order to present the data on H1N1 evolution in a more compelling manner, we included Figure *5*which shows the extensive mixing of isolates from different years in the phylogenetic tree of H1N1*.

#### Discussion section

I have some doubts about the interpretation of periods of "stasis" and of "rapid evolution". Could this distinction not be primarily due to incomplete nature of the sequence set, temporally and spatially? While there may be relative stasis locally among the strains that were sequenced in certain seasons in NY or NZ, using a "perfect" dataset (every single flu strain globally is sequenced) the authors would probably find "rapid evolution" somewhere at any given time? This then would raise the question of how predictable the "rapid evolution" would be, and how useful such analyses would be in predicting the dominant strains of the next season.

**Author response: ***As indicated above, this is an important issue but we believe it is, effectively, logically impossible to explain our conclusions by the incompleteness of the data (Discussion, 6*^th^*paragraph)*.

On the final conclusion of the authors that the common view of influenza virus evolution as a positive selection-driven process is incomplete

I am not convinced that the view of long periods of stasis punctuated by short periods of fitness described here is not primarily due to the use of a biased dataset. If the authors disagree with this opinion (which I guess they would) it would be good to discuss the issue of bias, rather than just waiving it.

*Author response: Yes, we do disagree, i.e., we do not see how the present results can be explained by biases in the data and we did include the relevant discussion (see the responses above)*.

I found the parallel amino acid replacements in different genetic lineages interesting.

The last point worth mentioning is that prediction of the predominant strain of the next influenza season is not the primary task of the influenza surveillance network that serves the WHO vaccine strain selection process. The primary task is to identify the emergence of strains that are antigenically distinct and (as the result) will dominate the next season. This way, the number of vaccine strain updates can remain limited, which is important because of cost, the interest of vaccine manufacturers, and perhaps vaccine efficacy. It is unnecessary to update the vaccine based on genetic changes that do not affect the antigenic properties of the virus.

**Author response: ***We certainly never claimed that it is necessary to change (update) the vaccine on the basis of sequence changes per se. The salient considerations are different: i) it might be possible to predict the future dominant strains by sequence comparison, ii) the HI test is not necessarily reflective of all relevant antigenic changes, so sequence analysis might help identify other such changes*.

### Reviewer's report 2

**David Krakauer**, The Santa Fe Institute

Review of "Punctuated interpandemic evolution of influenza A virus"

The recent availability of broadly sampled influenza genomes spanning the years 1995–2005 provides an opportunity for a systematic analysis of genetic trends correlated with the emergence of flu pandemics. In this paper, the authors adopting a phylogenetic framework, report a punctuated mode of virus evolution, in which diversity accumulates during periods of stasis and outbreaks are facilitated by selection against common strains. Epistasis among sites of the HA gene provide one explanation for the "neutral" delays attending new outbreaks, where multiple compensatory changes are required for fixation of a new adaptive variant. This paper nicely serves to illustrate that a constant rate model for microbial evolution, in which virulence is treated in terms of the independent contribution of amino acids in an epitope, coupled to a simple competitive exclusion principle based on replicative advantage, is too simple. Rather, new strains can be generated neutrally long before their advantages are felt in a new selective context, where this context shifts according the efficacy of host immunity to dominant epitopes and the rates of recombination events promoting antigentic shift. I find the research very interesting, but I do find, however, the final discussion of vaccine development a little gratuitous given the findings of the paper and perhaps an unnecessary appeal to the utility of basic research.

**Author response: ***We appreciate these insightful comments. However, the discussion of the implications of the findings on influenza evolution for vaccine development, in our view, has nothing to do with "an ...appeal to the utility of basic research". On the contrary, in the specific case of influenza, vaccine development and the basic trends in virus evolution are inextricably (and traditionally) linked, and not considering the implications of our findings would be strange. This being said, the corresponding part of the discussion was modified to make it less evaluative*.

#### Detailed remarks

1. I distrust the use of the term fitness in the abstract (end of the Results section) to explain dominance, as in this sentence, it provides no new information and sounds vaguely circular.

**Author response: ***Hard to agree. We believe that the current wording, indeed, describes the situation properly: due to the drop of the relative fitness of the previously dominant H3N2 isolates, the H1N1 isolates become dominant*.

2. The final sentence of the results section of the abstract could be stated more clearly. How about "The increase in dominance of H1N1 in some seasons is most likely facilitated by an increased resistance to the incumbent H3N2 lineage during a period of stasis."

**Author response: ***Again, we find the original wording to be more precise, even if slightly more complicated. The issue is, indeed, about the increase in the fraction of resistant hosts, not just "resistance" in general terms. The preceding sentence, though, has been reworded for clarity*.

3. The final sentence of the conclusion of the abstract is rather odd. How could parallel replacements serve as predictors of new dominant strains?

**Author response: ***Why not? The isolates that undergo parallel changes are likely to be doing so due to positive selection and might become dominant. This is explained in the Discussion. We do recognize, however, that this is speculative and the language in the Abstract has been softened accordingly*.

4. I do worry a little about the lack of statistical significance for some of the results, given that they are described as striking.

**Author response: ***Point well taken, "striking" removed*.

5. Figure 2 is rather impenetrable and could use a better explanation

**Author response: ***We added a new Figure *2*(such that the original Figure *2*became Figure *3) *that, hopefully, provides such a explanation, at least with regard to the procedure used to determine lineage extinction times*.

6. I wonder whether it is not appropriate to simulate (through bootstrapping for example) a null expectation for the pattern of recurrent amino acid substitutions in the data. It is hard to intuit how unlikely these events are.

**Author response: ***We felt that this was beyond the scope of the present work (it might become a subject of a future study). Indeed, the main point here is the non-random distribution of the parallel mutations across the lineages, and to establish this, simulation does not seem to be required*.

7. I am not yet convinced that what is being described is competition among strains (last paragraph of the **Results**). Competition implies a finite supply allocated over a potentially more abundant demand population, thereby promoting competitive exclusion. Here it is not clear that the immune suppression of one strain is what allows another to invade, or rather a simple suppression of one and an indifference to another. This would be more like sorting than selection in Wimsatts' usage with shifting indifference allowing outbreaks. Perhaps a little more detail on mechanism would help to make this distinction clear?

**Author response: ***There are some semantic issues involved here that we are not entirely confident about. Regardless, there does seem to be a competition (over a finite supply of susceptible individuals) between H1N1 and H3N2, the outcome of which depends on the fraction of the population that is naïve to each of the respective viruses at a given time and evolution of the viruses themselves that may allow them to escape immunity*.

8. In the **Discussion**, an increase in fitness is associated with rapid exploration of the adaptive landscape. But this is not really correct. Selection increases the rate of hill climbing to a local peak, but suppresses variance over the landscape as a whole. As the authors argue earlier in the paper, it is neutral evolution in the functionally "static" period that promotes effective search, then new variants are fixed through a rapid bout of selection.

**Author response: ***Yes, we must agree with this point. The phrase in question was modified to indicate that, under selection, only specific areas on the landscape are quickly explored*.

9. I think that the papers of Stadler and Fontana (e.g. current biology evodevo with RNA) on the role of neutrality in promoting innovation more suitable than Gould and Wagner.

**Author response: ***We now cite Huynen, Stadler and Fontana, in addition to Gould and Wagner, along with a relevant paper by one of us (Lipman and Wilbur 1991, ref*. [23]*)*.

10. A final remark on ds/dn ratios which offers an independent sites model of selection. Given the finding that epistasis is likely to be very important, I worry about the power of the ratio to reveal systematics trends. For example, there could be consistently strong directional selection, but little evidence of it, as a result of the compensatory changes required by epistasis. I guess when dn >> ds this might be sufficient to conclude directional selection, but when ds > dn we can not really know. This is hardly a unique failing of the current paper, but a common problem faced by the community of researchers intent on quantifying selection.

**Author response: ***The ambition of this paper is less general than *"quantifying selection". *What we seek to demonstrate and what does seem to become apparent is the existence of major differences in the strength of selection between different time intervals during influenza virus evolution*.

### Reviewer's report 3: Christopher Lee, University of California-Los Angeles

This is a very interesting paper that sheds new light on a scientifically and medically important question. It will certainly be of interest to a broad audience of readers of *Biology Direct*. The data and analysis methods appear sound, although I have some questions about the graphical presentation and interpretation of the results. I also suggest that additional text and a figure explaining some of the paper's definitions and methods would be useful.

1. It would be helpful to have more explanation of exactly what criteria were used for choosing the set of sequences for identifying mutations and measuring dn/ds ratios. The methods section indicates that only 100 H3N2 HA sequences (of the 994 available) were used to obtain dn/ds estimates. But the manuscript doesn't explain how the 100 sequences were chosen, why so few were used, and how robust the dn/ds results would be if other samples of HA sequences were used. If the results have some dependence on the sample chosen, it would be useful to give some measure of their robustness (e.g. bootstrap).

**Author response: ***We amended the text in question to indicate that the selected subsets represented all distinct side branches (i.e., all except some that were adjacent in the tree and were merged). It should be noted that the pattern of mutations mapped to the trunk branches and, accordingly, the dn/ds estimates for the trunk is highly robust to the specific choice of isolates from the side branches inasmuch as all of these branches are represented. Of course, the dn/ds estimate for the side branches themselves depends on the choice of isolates more strongly but this is not crucial for our conclusions. As for bootstrap or a similar test, this is, unfortunately, computationally prohibitive*.

2. Given that the authors are having trouble showing statistical significance for the dn/ds results, I'd assume the authors are using all the counts they can get; thus, some further explanation of limiting the analysis to "100 HA sequences" would be helpful. Similarly, do the mutations counted in Fig. 2 (and shown in Fig. 3) comprise ALL mutations in all 994 H3N2 HA sequences, or just a subset? Is this (sub)set of sequences different from that used for the dn/ds calculations, and if so why? Alternatively, if this (sub)set does not include all mutations observed in the 994 sequences, what is the rationale for this different choice of sequences? Reading the text I thought the dn/ds counts in Fig. 2 should be measured solely on trunk branches ("...positive selection in the epitope regions of each new dominant lineage..."), but the text doesn't explicitly say so. If the dn/ds numbers in Fig. 2 are not specific to the trunk branches, it would be helpful if the authors could address how these results really relate to the "dominant lineage" they refer to.

**Author response: ***See above. The maximum likelihood calculations of *dn/ds *were performed for a subset of 100 sequences for all branches and also for trunk and non-trunk branches separately as explained in the 3*^*rd *^*paragraph of the Results section and shown in Table *1. *As indicated in the response to (1), this gives a complete picture of the mutations in the trunk. The rest of the analysis dealt with all 994 sequences; in particular, the data in Figure *3*(former Figure *2) *includes all trunk mutations as is made explicit in the revised legend*.

3. Since the dn/ds differences report in paragraph 3 of the results ("To determine whether positive selection...") were not statistically significant, it might make sense to move this paragraph after the following paragraph (describing Fig. 2 and epitope vs. non-epitope Fisher tests), whose dn/ds differences were statistically significant. The current paragraph 3 could then be positioned as an extension of the statistically significant result, suggesting that there is indeed dn/ds>1 positive selection.

**Author response: ***We understand the potential advantages of this order of presentation but we feel that the current order is more logically cogent: the results of the standard test are given first; these are suggestive but not compelling, so additional, less common tests are described, and these do show that differences in dn/ds values are statistically significant*.

4. Distinguishing "trunk" vs. "non-trunk" branches is fundamental to much of the paper's analysis, but is not explained prominently (the definition is hidden in the Methods). Since the authors' criterion is quite simple, I think it should be explained prominently in the paper, at the first point these terms are used. I also think the definition of "dominant lineage" needs to be made explicit; e.g. does this always mean the trunk branch? Similarly, it would be helpful to clarify the distinction between "trunk branches" (column title in Table 1) vs. "inter-clade branches" (column title in Table 2).

**Author response: ***The definition of the trunk is included in the revised background section (4*^*th*^*paragraph). As for the "inter-clade" branches, this is used for H1N1 because there was no real trunk in that tree; this is made explicit in the revised footnote to Table *2.

5. The precise definition of how H3N2 isolates are divided into "lineages" is a bit hard to follow, and would be greatly clarified by an explanatory figure. The Methods section "Extinction of Lineages..." bristles with graphical language ("below", "above", "proximal", "distal" etc.), but all these complicated descriptions are not easy to picture. The authors seem to be going to considerable trouble to describe a picture to the reader; I think it would be much easier to just *show *the picture as a figure, with all the relevant terms defined and labeled on the figure. As far as I can tell, extinction times are computed for each trunk branch node, based on the isolation times of its non-trunk descendants, but a figure could make the definition both clear and precise. For example, does the "90% extinction" criterion mean sorting the non-trunk descendant isolates by date, and finding the 90^th ^percentile rank value of the isolate dates? The "trunk" vs. "non-trunk" definition (see above) could also be included in this figure. Since *Biology Direct *has no limit on pages or figures, this seems like an easy solution. I'm hoping this would also clarify Fig. 2 (see next point).

**Author response: ***The procedure employed to estimate extinction times is now illustrated in the new Figure *2; *we believe that this does, indeed, facilitate understanding of Figure *3*(former Figure *2).

6. In Fig. 2, why are extinction times shown for some trunk branch nodes, but not others? E.g. between the upper "41" and the "4" right above it, there are many trunk branch nodes, but extinction times are not shown for them. What is the criterion that determines which trunk branch nodes have extinction times computed for them?

**Author response: ***The extinction times are given for all segments of the trunk bounded by precisely dated isolates. Without such dating, extinction time could not be estimated. This is noted in the last section of the Methods*.

7. In Fig. 2, it would be helpful to label the left column "Extinction time (mo.)" and the right column "Mutations (n/s)".

**Author response: ***done as suggested*.

8. In Fig. 3, the use of color is potentially confusing (i.e. red/green means very different things for the lines vs. letters). I suggest that all mutations simply be printed in black, with BOLD for epitope mutations, and ITALICS for non-epitope mutations.

**Author response: ***modified as suggested*.

9. Comparing the amino acid mutation counts in Fig. 2 vs. the mutations shown in Fig. 3, the numbers don't match, so it would be useful for the authors to clarify precisely the criteria for what mutations are included in the counts for Fig. 2 vs. Fig. 3. I had assumed that the Fig. 2 counts would only reflect trunk branches (to measure positive selection on the "dominant", trunk branch); am I wrong?

*Author response: These figures (*3* and *4* in the revision) present different data. Only those mutations in the trunk for which there are parallel mutations in side branches are shown in Figure *4. *Thus, there is no reason to expect the same numbers*.

10. It might be possible to improve the presentation of the data in Fig. 2. Specifically, we're asked to correlate two important types of data in this figure (extinction times and dn/ds numbers); both are quantitative, but are only presented as text, and are disconnected from each other by two layers of "mapping". I suggest that this be reformatted to show two separate results: the raw data for the extinction time result, and the correlation of extinction times vs. dn/ds mutation events.

The first reason for suggesting this is that the extinction time result is in fact an important result in itself, and I think readers of the current manuscript will have trouble understanding exactly how the authors got this result. I suggest the following graphic. Use the y-axis to represent the origination date of trunk branch nodes (i.e. inferred origination date of each lineage, with a point shown for each lineage), essentially drawn just like the time-axis currently shown on the right hand side of Fig. 2. Use the x-axis to show extinction times: draw a horizontal bar showing the extinction time for each lineage; optionally, you could also draw tick marks showing the raw isolate dates (relative to the lineage origination date). Hopefully this would allow readers to evaluate for themselves the raw data for the existence of two distinct phases of "stasis" vs. "rapid turnover". It would also be helpful to calculate some kind of p-value for this hypothesis, and to show a histogram of lineage extinction times so people can see "two peaks" (or at least, not one peak).

There are several options for showing the correlation vs. mutation counts. The mutation counts could simply be juxtaposed as text (as in the current Fig. 2). Additionally, dates of individual mutation events (epitope, non-epitope, and synonymous, each with a distinct symbol) could be marked on the right hand side based on their inferred date, so that readers can see the raw data. Doing this as a separate figure (i.e. not within Fig. 2) would be OK, if that helps reader comprehension by letting the reader focus on one result at a time.

**Author response: ***We tried several alternatives to Figure *3* (former Figure *2*) but, in the end, decided to stick to the original format. Hopefully, the new Figure *2*does clarify at least some of it (also see the response to the next point)*.

11. Is it possible to include in the Fig. 2 the dominance period of each subtype? This would make it easy to see whether the H3N2 stasis is associated with H1N1 dominance or not.

**Author response: ***As per this suggestion, the H1N1 dominance intervals are now shown*.

12. For a general audience, it would be helpful to add some explanation of what "Immune cross-protection" means, e.g. "low frequency of co-infection by both H1N1 and H3N2"

**Author response: ***In this context, immune cross-protection simply means that individuals that develop immune response to H1N1 are less likely to get sick when infected with H3N2 and vice versa. Co-infection is not involved*.

I hope some of these questions are helpful.

## Authors' contributions

YIW performed the bulk of the analysis and contributed to the interpretation of the results, CV performed the analysis of the epidemiological data, ECH performed parts of the analysis of selective pressures, EVK contributed to the interpretation of the results and writing of the manuscript, DJL initiated the study, contributed to the interpretation of the results and wrote the initial draft of the manuscript; all authors edited and approved the final version of the manuscript.

## Supplementary Material

Additional file 1 Multiple alignment of influenza virus sequences. Multiple alignments of H3N2 HA, H3N2 PA and H1N1 HA coding sequences and H3N2 HA amino acid sequences with antigenic epitopes mapped in aligned FASTA format. Click here for file
